# Implementation of “COVID-19 case investigation and contact tracing” training program in Armenia

**DOI:** 10.1093/eurpub/ckac131.038

**Published:** 2022-10-25

**Authors:** Z Grigoryan, L Musheghyan, D Abrahamian, V Petrosyan, A Dorian

**Affiliations:** 1 Turpanjian College of Health Sciences, American University of Armenia, Yerevan, Armenia; 2 Fielding School of Public Health, University of California Los Angeles, Los Angeles, USA

## Abstract

**Background:**

Comprehensive contact tracing and case identification (CICT) is key to preventing the SARS-COV-2 transmission. During the COVID-19 waves in Armenia, the effectiveness of CICT practices was suboptimal due to weak capacity of evidence-based CICT. The American University of Armenia and the University of California, Los Angeles offered a continuous education program for the public health workforce in Armenia which is relevant for any country aiming to improve its CICT capacities.

**Objectives:**

We developed and implemented a fully remote yet synchronous ‘COVID-19 case investigation and contact tracing” training program through didactic lectures, demonstrations of CICT interviews, small-group skill labs, and opinion polls. It covered the basic principles of public health and epidemiology, the main methods of CICT in relation to COVID-19, and health coaching techniques. We evaluated the knowledge improvement through a pre-experimental evaluation design at baseline and follow-up. The training sessions were held from November 2020 to June 2021. The participants received 10 CME credits upon completion of the training course.

**Results:**

The online modality allowed to reach professionals across the country, though affected the participation in the evaluation surveys. Overall, 93 professionals participated in the training program, yet only 57 returned completed surveys. The paired analysis showed an increase in the mean knowledge scores (0-24) at baseline and follow-up (14.5 vs 15.82 (p = 0.0083). A more notable increase was detected on questions that measured knowledge of health coaching techniques (0-6) (2.76 vs 3.44 (p = 0.0052)).

**Conclusions:**

New knowledge and skills penetrated into daily CICT practices across Armenia. This was a good example of a quick mobilization of local and international expertise to assist the national efforts in responding to a public health emergency by utilizing evidence-based approaches and lessons learned from the past infectious diseases’ outbreaks.

**Key messages:**

• Our training program strengthened the human resource capacities of Armenia’s existing public health system and assisted Armenia’s COVID-19 response efforts.

• The model of our training program can be effectively adapted and extrapolated for various countries and settings which aim to improve response capacities to other threats of public health importance.

